# Biomechanical Analysis of a Novel Double-Point Fixation Method for Displaced Intra-Articular Calcaneal Fractures

**DOI:** 10.3389/fbioe.2022.791554

**Published:** 2022-03-09

**Authors:** Miko Lin Lv, Ming Ni, Wanju Sun, Duo Wai-Chi Wong, Shuren Zhou, Yongwei Jia, Ming Zhang

**Affiliations:** ^1^ School of Medical Instrument and Food Engineering, University of Shanghai for Science and Technology, Shanghai, China; ^2^ Department of Orthopedics, Pudong New Area People’s Hospital Affiliated to Shanghai Jiaotong University, Shanghai, China; ^3^ Department of Biomedical Engineering, Faculty of Engineering, The Hong Kong Polytechnic University, Kowloon, Hong Kong SAR, China; ^4^ School of Computer and Communication Engineering, Changsha University of Science and Technology, Changsha, China; ^5^ Department of Spine Surgery, Guanghua Hospital, Shanghai University of Traditional Chinese Medicine, Shanghai, China

**Keywords:** internal fixation, volar distal radial plate, calcaneal locking plate, finite element method, displaced intra-articular calcaneal fracture

## Abstract

The development of minimally invasive procedures and implant materials has improved the fixation strength of implants and is less traumatic in surgery. The purpose of this study was to propose a novel “double-point fixation” for calcaneal fractures and compare its biomechanical stability with the traditional “three-point fixation.” A three-dimensional finite element foot model with a Sanders type IIIAB calcaneal fracture was developed based on clinical images comprising bones, plantar fascia, ligaments, and encapsulated soft tissue. Double-point and three-point fixation resembled the surgical procedure with a volar distal radius plate and calcaneal locking plate, respectively. The stress distribution, fracture displacement, and change of the Böhler angle and Gissane’s angle were estimated by a walking simulation using the model, and the predictions between the double-point and three-point fixation were compared at heel-strike, midstance, and push-off instants. Double-point fixation demonstrated lower bone stress (103.3 *vs.* 199.4 MPa), but higher implant stress (1,084.0 *vs.* 577.9 MPa). The model displacement of double-point fixation was higher than that of three-point fixation (3.68 *vs.* 2.53 mm). The displacement of the posterior joint facet (0.127 *vs.* 0.150 mm) and the changes of the Böhler angle (0.9° *vs.* 1.4°) and Gissane’s angle (0.7° *vs.* 0.9°) in double-point fixation were comparably lower. Double-point fixation by volar distal radius plates demonstrated sufficient and favorable fixation stability and a lower risk of postoperative stress fracture, which may potentially serve as a new fixation modality for the treatment of displaced intra-articular calcaneal fractures.

## Introduction

Displaced intra-articular calcaneal fractures (DIACFs) are highly disabling injuries usually caused by high-energy trauma (e.g., fall from height) and represent treatment challenges to orthopedic surgeons (Dhillon and Prabhakar, 2017). Recovery after treatment is often prolonged, with poorer functional results compared with other orthopedic conditions (van Tetering and Buckley, 2004). Thus, restoration to preinjury level of functioning is unlikely (Ibrahim et al., 2007). Conservative interventions were once recommended and demonstrated to provide comparable outcomes with operative treatments (Ibrahim et al., 2007). However, several studies have shown that nonoperative interventions often lead to delays in the reconstruction of deformed fractures, leaving patients with painful, stiff feet and permanent disability (Bruce and Sutherland, 2013). Since the mid-1990s, surgical treatment using open reduction and internal fixation (ORIF) was advocated and became the standard treatment for DIACFs, producing anatomical reduction and satisfactory functional outcomes (Sanders, 2000).

**FIGURE 1:**
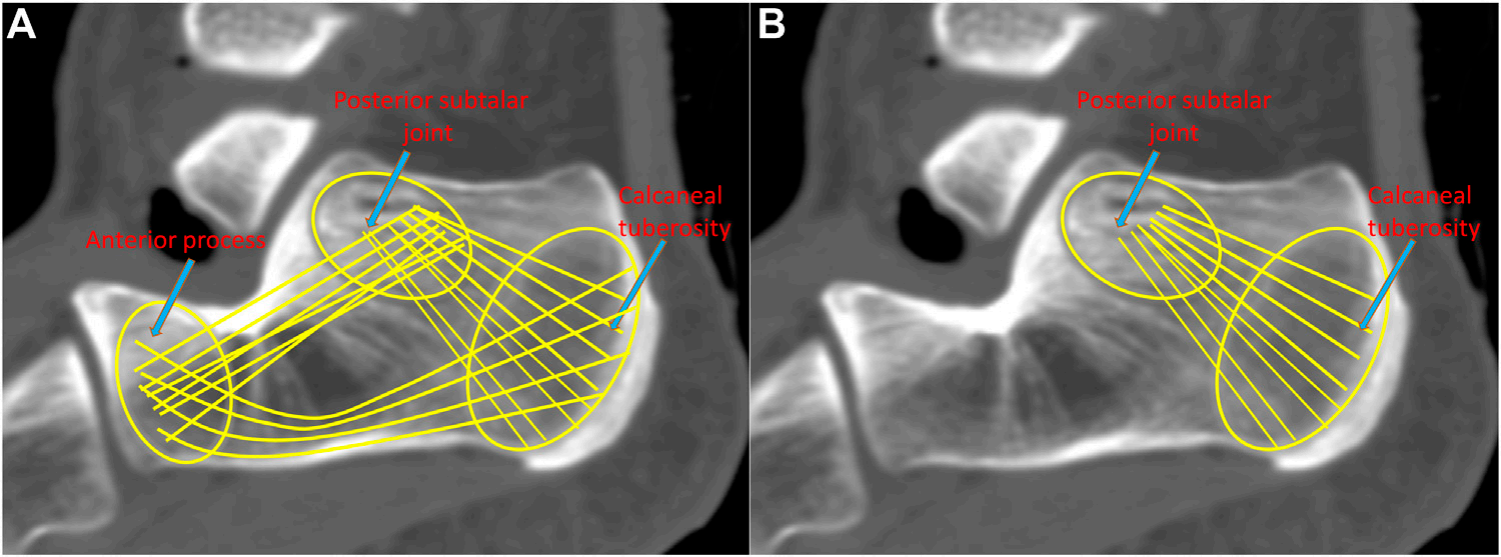
Demonstration of the three-point fixation (TPF) **(A)** and the double-point fixation (DPF) **(B)** mechanisms.

ORIF aims to achieve reliable stability by securing three-point fixation (TPF) (Figure 1A), i.e., the anterior process, posterior subtalar joint, and calcaneal tuberosity, at the cost of a large incision (Chen et al., 2017). The calcaneal locking plate has been widely adopted in ORIF since it can provide sufficient fixation, as well as a relatively lower risk to the devascularization of fragments (Mostafa et al., 2010). However, ORIF results in a high complication rate of approximately 30% and predisposes patients to serious comorbidities (Shah and Parmar, 2020). Therefore, there is growing interest in developing alternative methods with smaller incisions to minimize soft tissue trauma and resulting complications. Minimally invasive fixation (MIF), which compromises adequate visualization, has emerged and is used increasingly. Shah and Parmar (2020) evaluated MIF *via* the sinus tarsi approach using locking recon plates, demonstrating that MIF led to satisfactory clinical outcomes and a reduced rate of soft tissue-related complications. In addition, Ene et al. (2013) documented the outcome of MIF using the Essex-Lopresti osteosynthesis technique with no reported cases of postoperative wound infection or skin necrosis. In a recent report, the biomechanical characteristics of MIF and ORIF were found to have similar stability performance (Zhang et al., 2020).

MIF could also be achieved by the so-called double-point fixation (DPF) (Figure 1B), i.e., the posterior subtalar joint and calcaneal tuberosity. Simon et al. (2015) proposed a locking nail for the treatment of displaced articular fractures through fixed posterior subtalar joint and calcaneal tuberosity. Biomechanical and clinical studies have shown that DPF can reposition the subtalar joint well and satisfy the clinical results (Goldzak et al., 2014). We believe that DPF can also be facilitated by the volar distal radius plate. The slight thickness (1.5 mm) of the plate is advantageous in reducing the risk of soft tissue irritation and infection. The length of the plate is similar to that of the calcaneal tuberosity, and the curved T-shaped design facilitates fixation for the subtalar joint and calcaneal tuberosity. The short limb can fix the posterior subtalar joint with five locking screws, and the long limb can fix the calcaneal tuberosity with three screws. As a proof of concept, it is necessary to know whether the DPF can substantiate sufficient fixation strength for DIACF and not induce exceeding stress.

To this end, the objective of this study was to evaluate the biomechanical performance (in terms of stress distribution, fracture displacements, Böhler angle, and Gissane’s angle) of DPF and compare it to that of TPF. We hypothesized that the biomechanical stability of DPF was comparable to that of TPF and thus satisfied the biomechanical demands for secure fixation and could facilitate early weight-bearing rehabilitation. Given a proper model geometry and a set of material properties and boundary conditions, finite element (FE) analysis can provide valuable biomechanical information on the internal environment of the model parts under a preset simulated scenario (Zanetti and Bignardi, 2013; Galbusera et al., 2014; Pascoletti et al., 2018; Scarton et al., 2018; Ün and Çalık, 2019). The evaluation was carried out using FE analysis, which provides a multifunctional platform to evaluate the internal biomechanical environment of the human body and has been widely used to evaluate surgical outcomes, the pathomechanics of surgical complications, and implant design (Wang et al., 2016). In addition, the FE model also facilitates wider investigations of foot deformity, trauma, and rehabilitation (Wang et al., 2016).

## Materials and Methods

### Model Reconstruction

This study was approved by the Ethical Committee of the hospital (no. 2019-16). A 63-year-old female health participant (height, 161 cm; body weight, 64 kg) was recruited as the model participant. Computed tomography (CT) and magnetic resonance imaging (MRI) were performed on the left foot and ankle of the model participant using slice intervals of 1 and 1.25 mm, respectively. The images were integrated and segmented in medical imaging processing software (Mimics 15.0, Materialise, Leuven, Belgium) and reverse engineering software (Geomagic 2015, 3D Systems, Rock Hill, United States), through which the geometry of the foot model was reconstructed.

**FIGURE 2:**
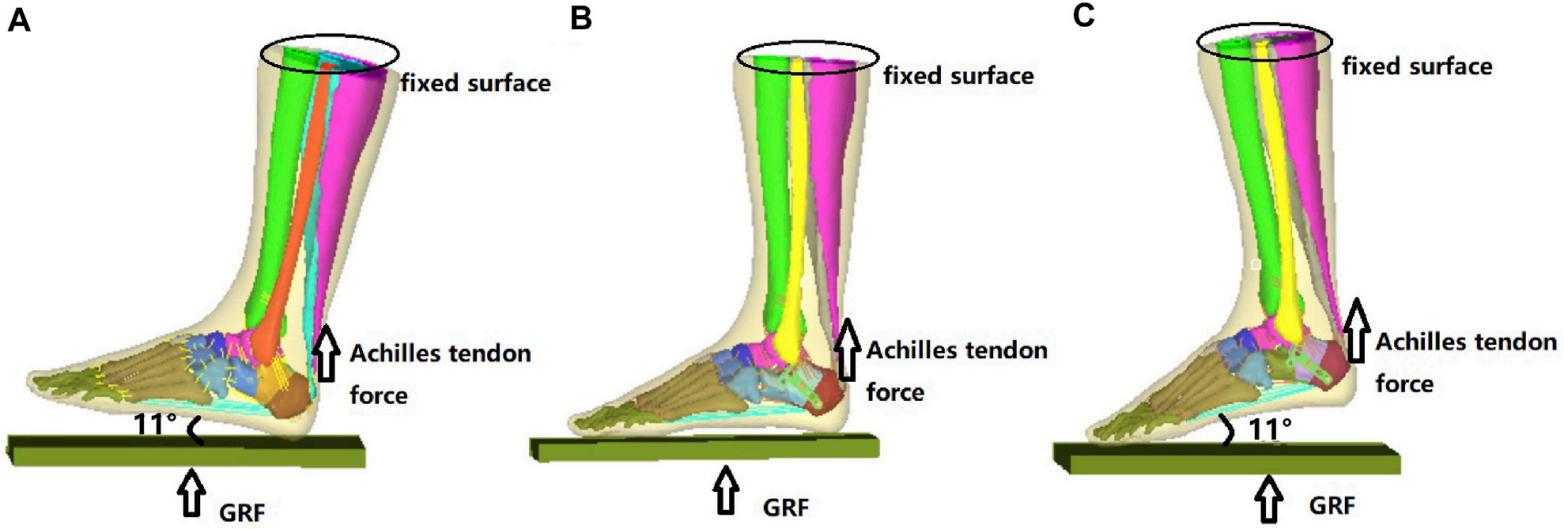
Reconstructed model of the foot-and-ankle complex. **(A)** Reconstructed model geometry. **(B)** Finite element (FE) model after mesh creation. **(C)** Enlarged model view of the double-point fixation (DPF). **(D)** Enlarged model view of the three-point fixation (TPF) (1, anterior process; 2, lateral part of posterior subtar joint; 3, middle part of posterior subtalar joint; 4, sustentaculum tali; 5, calcaneal tuberosity; *x*, medial; *y*, anterior; *z*, superior; GRF, ground reactiono force).

As shown in Figure 2A, the reconstructed model geometry consisted of 26 bones (tibia, fibula, talus, calcaneus, navicular, cuboid, 3 cuneiforms, 5 metatarsals, and 14 proximal and distal phalanges), in addition to the triceps surae (gastrocnemius and soleus), ligament, plantar fascia, and encapsulated soft tissues. A Sanders type IIIAB fracture resembled the reconstructed foot and ankle model by creating fracture gaps of 0.1 mm (Goldzak et al., 2012), which separated the calcaneus into five sections: the anterior process fragment, the sustentacular fragment, the middle and lateral fragments of the posterior subtalar joint, and the tuberosity fragment, as shown in Figure 2B.

Two model conditions were created in response to the two surgical conditions. The geometry of the two implants (volar distal radial plate and calcaneal locking plate) was constructed according to the manufacturers’ specifications (Depuy Synthes, Raynham, PA, United States) using SolidWorks computer-aided design software (Dassault Systèmes, Vélizy-Villacoublay, France). For DPF, the volar plate was fixed on the posterior joint fragment and calcaneal tuberosity with five 2.4-mm locking screws (S1–S5) and three 2.7-mm locking screws (S6–S8), respectively, as shown in Figure 2C. For TPF, the calcaneal plate was fixed onto the anterior fragment (S1–S4), the subtalar joint (S5–S8), and the calcaneal tuberosity (S9–S12) by 3.5-mm locking screws, as shown in Figure 2D.

### Mesh Creation

The model geometry was exported to Hypermesh 13.0 (Altair, Troy, MI, United States) for mesh creation. Both the plantar fascia and ligament were meshed as two-node truss units, while other parts of the lower limb were assigned four-node three-dimensional distributional stress tetrahedron units (C3D4). For ground, we meshed it with an 8-node reduced integrated hexahedral element (C3D8R).

The FE model of the intact foot comprised 80,311 nodes, 455,104 elements, and 137 truss units. The DPF model comprised 157,672 nodes, 762,929 elements, and 137 truss units, while the TPF model comprised 161,137 nodes, 773,437 elements, and 137 truss units.

### Material Properties

As shown in Table 1, the material properties were idealized as homogeneous, isotropic, and linearly elastic for the bone, plantar fascia, and ligaments, according to the data from a relevant review (Morales-Orcajo et al., 2016). The hyperelastic material of the encapsulated soft tissue (Morales-Orcajo et al., 2016) and muscle bulk tissue of the gastrocnemius–soleus (Reeves et al., 2005; Yamamura et al., 2014) was specified using a second-order polynomial strain energy potential equation. The plates and screws were assigned a titanium alloy material. None of the bones, soft tissues, or the implant components were pre-strained ***.***

**TABLE 1:** Material properties used in the finite element model.

Parts	Young’s modulus, *E* (MPa)	Poisson’s ratio, *v*	Density (kg/m^3^)	Cross-sectional area (mm^2^)
Bone	7,300	0.3	1,500	–
Plantar fascia	350	–	937	58.6
Ligament	260	–	937	18.4
Titanium	110,000	0.3	4,540	–
Ground	17,000	0.1	5,000	–
Outer encapsulated soft tissue	Hyperelastic material property with second-order polynomial strain energy potential equation coefficients			
	*C*_10_ = 0.08556 N mm^−2^, *C*_01_ = −0.05841 N mm^−2^, *C*_20_ = 0.039 N mm^−2^, *C*_11_ = −0.02319 N mm^−2^, *C*_02_ = 0.00851 N mm^−2^, *D*_1_ = 3.65273 mm^2^ N^−1^			
Muscle tissue (gastrocnemius, soleus, and Achilles tendon)	*C*_10_ = 8.57000 N mm^−2^, *C*_01_ = 12.1000 N mm^−2^, *C*_20_ = 936.000 N mm^−2^, *C*_11_ = 718.000 N mm^−2^, *C*_02_ = 480.000 N mm^−2^, *D*_1_ = 0.00413 mm^2^ N^−1^			

### Boundary and Loading Conditions

**FIGURE 3:**
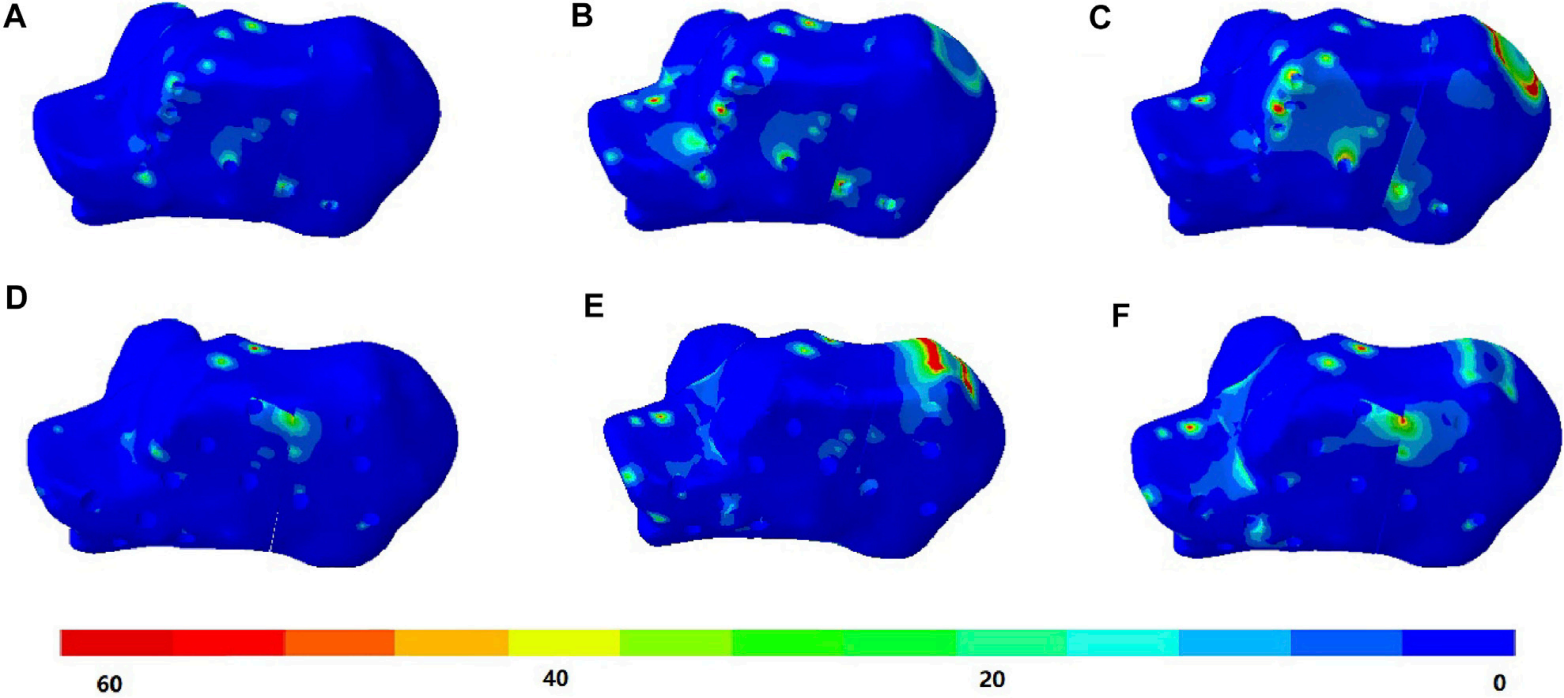
Boundary and loading conditions of the finite element (FE) model at heel-strike instant **(A)**, midstance instant **(B)**, and push-off instant **(C)**. *GRF*, ground reaction force.

Three gait instants (i.e., heel strike, midstance, and push-off) were simulated in the FE model. As shown in Figure 3, the superior surface of the shank was fixed proximally. Vertical ground reaction forces (GRFs) were applied on the ground plate, which were 704, 608, and 736 N for the three walking stance instants, respectively (Chen et al., 2015; Yu et al., 2016). The magnitude of GRFs corresponded to 110%, 95%, and 115% of body weight. The Achilles tendons were 480, 550, and 1,100 N for the three walking stance instants, respectively (Gefen et al., 2000; Fröberg et al., 2009; Arnold et al., 2010) and were applied at the insertion point of the triceps surae at the calcaneus. The coefficients of friction between the contact pairs ground-to-encapsulated soft tissue, bone fragment-to-screws, and bone fragment-to-bone fragment were 0.6, 0.3, and 0.3, respectively (Dai et al., 2006; Bulaqi et al., 2015). The screws were tied to the plate, and there was no interaction between the bone and the plate. The FE simulation was performed in the commercial FE software package Abaqus (Dassault Systèmes, Vélizy-Villacoublay, France).

### Outcome Measures

The primary outcome included the maximum von Mises stress of the calcaneus and implants, in addition to the joint facet displacement, the Böhler angle, and Gissane’s angle in three time instants. High stress of the calcaneus and implants implicated the risk of bone breakdown and implant failure, respectively, while the facet displacement and angle measures manifested fracture site stability and the risk of nonunion or malunion.

To measure Böhler’s angle, one line was drawn from the highest point of the tuberosity to the highest point of the posterior facet. Another line was drawn from the highest point of the anterior process to the highest point of the posterior facet of the calcaneus. Böhler’s angle was measured by the angulation of these two lines. Gissane’s angle was measured by the angulation between a line along the posterior facet of the calcaneus and another line drawn from the anterior process to the sulcus calcaneus. For the measurement of joint facet displacement, we first selected 10 pairs of nodes from two sides of the fragments according to a protocol (Ni et al., 2016; Ni et al., 2019). The coordinates of the nodes were recorded to calculate the average distance between the 10 pairs of nodes. The displacement of the joint facets was defined as the difference in the average distance before and after load bearing.

## Results

### FE Model Validation

The model has been validated previously by comparing the plantar pressure distribution of the FE prediction with that measured on the model participant in the existing study (Zhang et al., 2020). The current model essentially has the same model geometry and loading case as that of our existing model, with the exception of the surgery and implant. Given that the deviations in the validation metrics between the FE prediction and measurement were less than 5%, we believed that the FE analysis demonstrated good agreement with the physical measurement and was considered adequately reliable (Wong et al., 2021).

### von Mises Stress of Calcaneus

**FIGURE 4:**
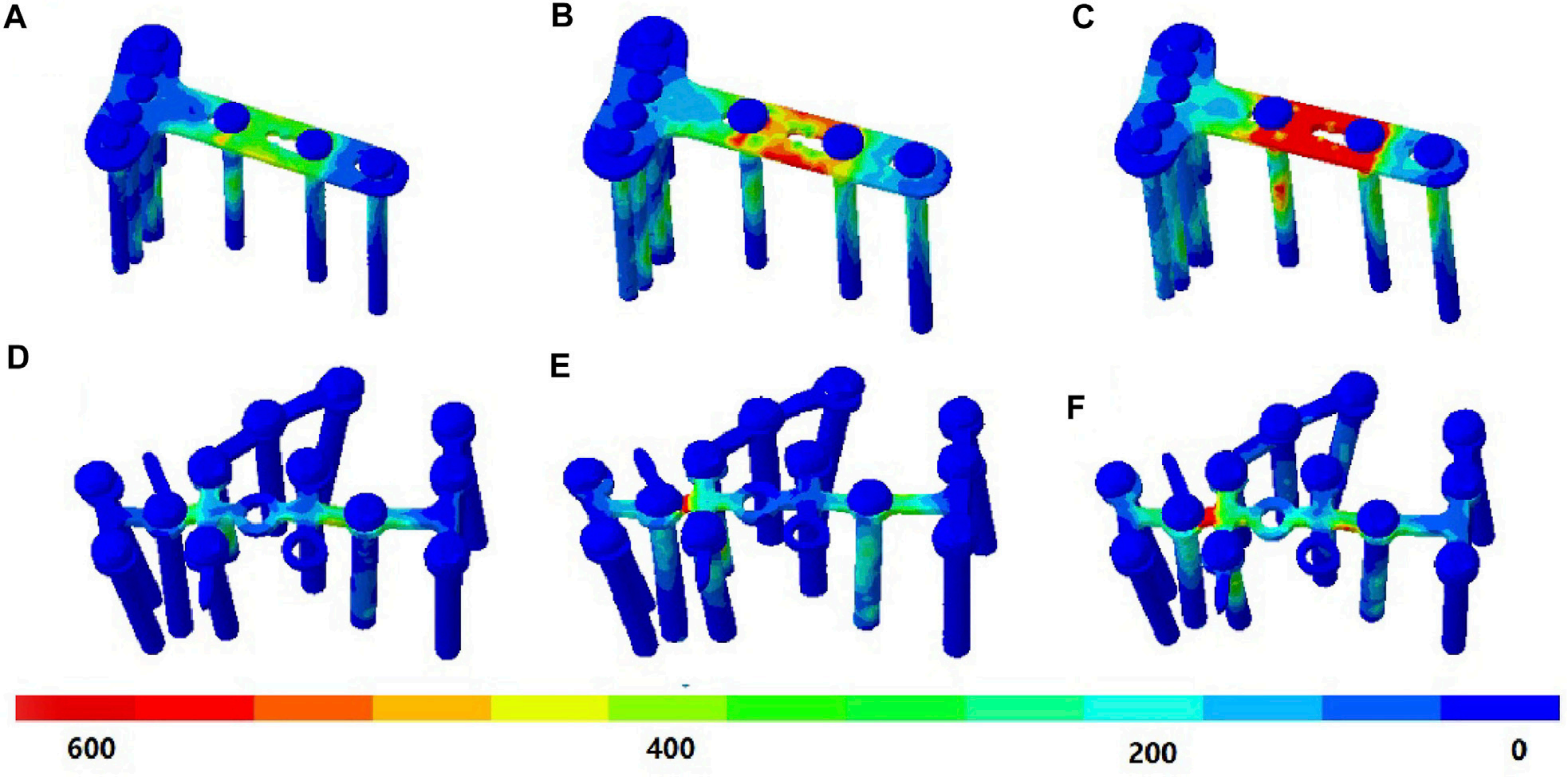
von Mises stress (in megapascal) of the calcaneus bone. **(A)** Condition at heel-strike instant. **(B)** Double-point fixation (DPF) at midstance instant. **(C)** DPF at push-off instant. **(D)** Three-point fixation (TPF) at heel-strike instant. **(E)** TPF at midstance instant. **(F)** TPF condition at push-off instant.

The stress distributions of the DPF and TPF at the heel-strike, midstance, and push-off instants are shown in Figure 4. Stress was mainly concentrated at the screw-to-bone interface and in the adjacent soft tissue. The maximum stresses of the calcaneus under DPF were 59.4, 92.7, and 103.3 MPa in the three instants, respectively, which were comparatively lower than those under the TPF condition (121.8, 163.0, and 199.4 MPa), as shown in Table 2.

**TABLE 2:** Maximum von Mises stress of the calcaneus and implants under different surgical conditions at different simulated time instants.

Part/time instant	Heel strike (MPa)	Midstance (MPa)	Push-off (MPa)
Volar plate	427.4	605.9	1,084.0
Calcaneal plate	210.3	411.0	577.9
Calcaneus under volar plate fixation (DPF)	59.4	92.7	103.3
Calcaneus under calcaneal plate fixation (TPF)	121.8	163.0	199.4

DPF, double-point fixation; TPF, three-point fixation

### von Mises Stress of Implants

**FIGURE 5:**
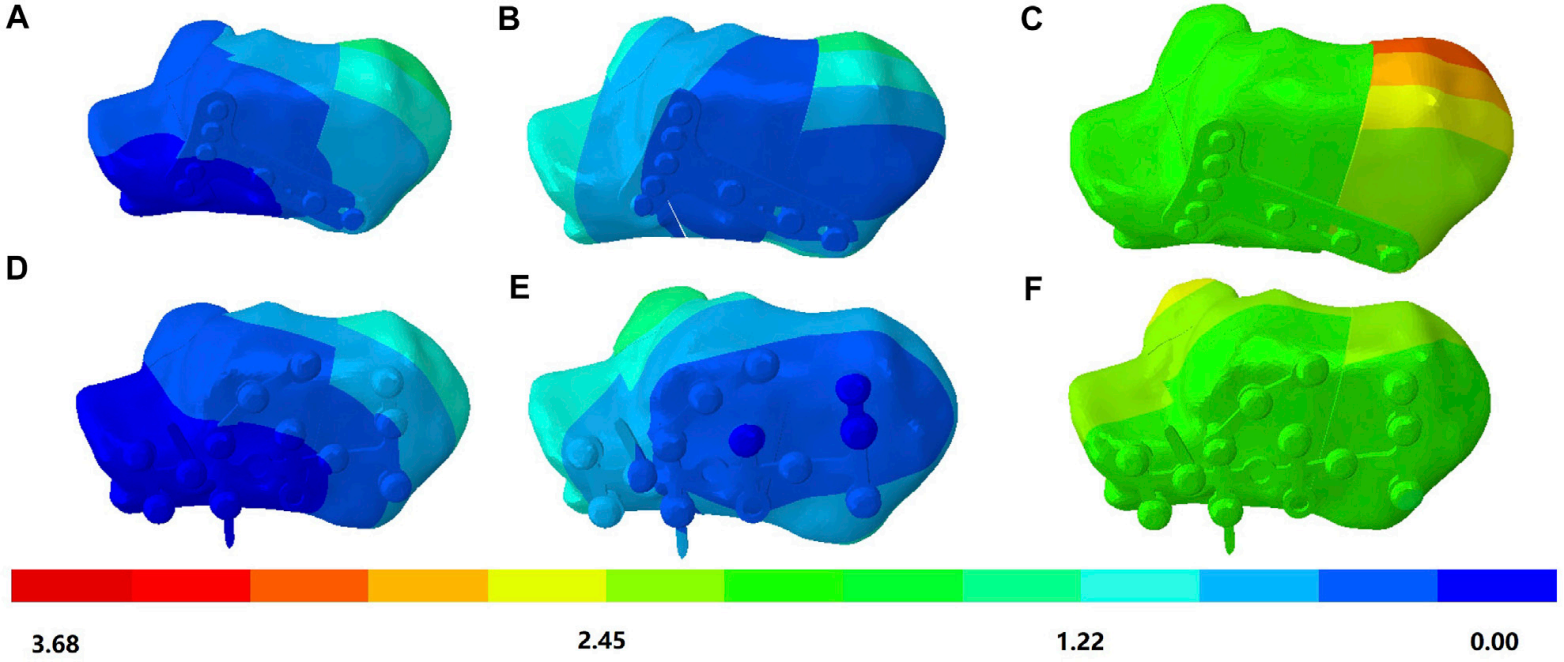
von Mises stress (in megapascal) of the implant. **(A)** Double-point fixation (DPF) at the heel-strike instant. **(B)** DPF at the midstance instant. **(C)** DPF at the push-off instant. **(D)** Three-point fixation (TPF) at the heel-strike instant. **(E)** TPF at the midstance instant. **(F)** TPF at the push-off instant.

Under the DPF, stress was concentrated in the middle of the long limb, which was located between the two screws, as shown in Figures 5A–C. Under the TPF, stress appeared to be more concentrated at the joint of the three limbs (Figures 5D–F). As listed in Table 2, the maximum stresses of the volar plate were 427.4, 605.9, and 1,084.0 MPa at the heel-strike, midstance, and push-off instants, respectively, which were comparatively larger than those of TPF (210.3, 411.0, and 577.9 MPa).

### Reduction and Fixation Conditions

**FIGURE 6:**
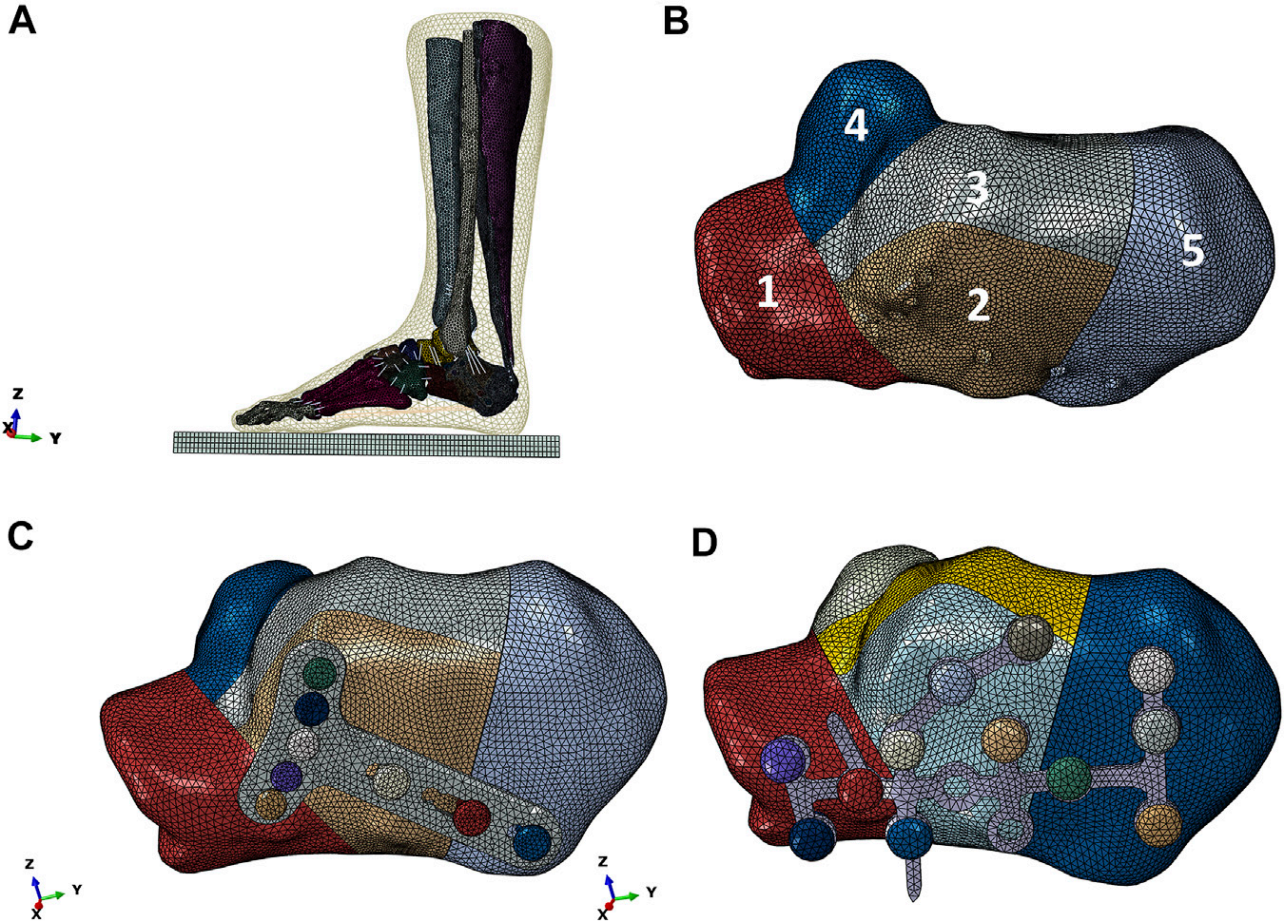
Magnitude of displacement (in megapascal) of the calcaneus and implant simulated with double-point fixation (DPF) at heel-strike instant **(A)**, midstance instant **(B)**, and push-off instant **(C)** and simulated with three-point fixation (TPF) at heel-strike instant **(D)**, midstance instant **(E)**, and push-off instant **(F)**.

As shown in Figure 6, for the DPF, the calcaneus displacement occurred maximally at the internal side of the calcaneal tuberosity and reached a peak value at the push-off instant, which was 3.68 mm. However, the TPF had a maximum displacement of 2.53 mm at the calcaneal tuberosity during the heel strike, which was transferred to the sustentaculum tali during the midstance and push-off instants.

The displacements of the posterior joint facet of the DPF were 0.076, 0.048, and 0.127 mm at the heel-strike, midstance, and push-off instants, respectively, which were less than those of the TPF (0.160, 0.060, and 0.150 mm).

The Böhler angle and Gissane’s angle were 31.8° and 128.5°, respectively, in the intact calcaneal model. Both fracture models showed that the Böhler angle decreased while Gissane’s angle increased. For both DPF and TPF, the Böhler angle and Gissane’s angle could meet normal requirements (Su et al., 2013), although MIF demonstrated more change, as shown in Table 3.

**TABLE 3:** Posterior joint facet displacement and angles in different surgical conditions.

Parameter	Condition	Heel strike	Midstance	Push-off
Total displacement (mm)	DPF	1.60	1.90	3.68
	TPF	1.22	1.61	2.53
Posterior joint facet fracture displacement (mm)	DPF	0.076	0.048	0.127
	TPF	0.160	0.060	0.150
Böhler angle (°)	DPF	31.6	31.2	30.7
	TPF	31.4	30.5	30.0
Gissane’s angle (°)	DPF	129.2	129.6	129.9
	TPF	129.9	130.5	130.7

DPF, double-point fixation; TPF, three-point fixation

## Discussion

DIACF is the most prevalent mode of calcaneus fracture, accounting for approximately 75% of cases (Marouby et al., 2020). The fracture pattern was classified using the Sanders classification based on the number of fracture lines (Sanders et al., 1993). The fracture lines of DIACF often occur anterior to the posterior joint facet and run through the middle of the calcaneal tuberosity that continues with the upper longitudinal lines and anterior transverse lines (Ni et al., 2021). In this study, we proposed a novel surgical option using a volar plate in the DPF procedure to treat DIACFs. As a proof of concept, a validated FE model of the foot–ankle–shank complex was constructed to evaluate the biomechanical performance of the implant and compare it to that of TPF. The strength of this study was that the reconstructed model considered the geometry of the bones, muscles, and encapsulated soft tissue, while some studies simplify these structures (Gu et al., 2015; Ouyang et al., 2017). As such, the simulation could be comparatively more comprehensive and reliable.

According to our FE predictions, DPF demonstrated adequate stability in treating DIACFs. Firstly, the changes in the Böhler angle and Gissane’s angle in the three walking stance instants were small and were within the reported normal range of the calcaneus (Su et al., 2013). Secondly, the maximum displacement of the posterior joint facet was 0.127 mm, which indicated that joint surface collapse and degeneration were unlikely (Potter and Nunley, 2009). Lastly, the maximum stress induced on the calcaneus was 103.3 MPa, which was lower than the yield strength of the bone (Frost, 1997). All these findings supported that there is a low risk of stress fracture and that early weight-bearing rehabilitation is likely possible. On the other hand, the use of volar plates provides several advantages. The small size could facilitate MIF, which decreases incision-induced complications. Moreover, most surgeons are familiar with the volar plate and the surgical procedures, meaning no additional training is required. Wong et al. (2015) commented that interfragmentary compression should be facilitated for healing and to avoid nonunion. In this study, we endeavored to quantify union or nonunion conditions by the displacement of the joint facet, while contact stress between the fragments could be an alternative to demonstrate whether interfragmentary compression could be achieved.

The mechanism of DPF coincided with the anatomic and physiological characteristics of the calcaneus. The calcaneus is the largest tarsal bone, with a cortical shell outside and trabecular bones inside. The loading pattern of the calcaneal trabecular structure could be categorized into four groups: the principal compressive group, the principal tensile group, the secondary compressive group, and the secondary tensile group (Gefen and Seliktar, 2004). The principal compressive trabeculae play a major role in resisting the stresses contributed by muscle forces and body weight. It sits on the posterior half of the calcaneus and extends from the posterior facet joint toward the calcaneal tuberosity (Saers et al., 2020), which is the point of fixation in DPF. Inserting the DPF along with the trabecular orientation could maintain the alignment and structure of the principal compressive trabeculae and, thus, could facilitate fracture union and functional recovery.

A previous study showed that DPF using intramedullary nails could provide better stability than its counterparts. Goldzak et al. (2014) conducted calcaneus fixations using calcanail and lateral angular stable plates on cadavers and showed that calcaneus fixation produced threefold less subtalar joint displacement than plate fixation, indicating better stability. Our study was in accordance with the findings of Goldzak and colleagues. In our prediction, DPF using a volar plate induced fewer changes in the Böhler angle and Gissane’s angle, as well as the displacement of the posterior joint facet, than TPF. This result could be attributed to the structure of the volar plate, which could fix the posterior subtalar joint by up to five screws. In the mechanism of DPF, the anterior process of the calcaneus was not fixed, and the reduction was maintained by the interfragmentary compression produced by the plantar fascia and intrinsic foot muscles. DPF could be a better option for fixation supported by the minimal change in critical angles.

Posterior joint facet fracture displacement and implant stress were two important factors that manifested fracture stability and implant failure, respectively. From our FE results, the stress distribution of DPF and TPF demonstrated observable differences. The implant design could be optimized by widening or thickening positions with high stress. A double-support design or additional screws can also be adopted to share the mechanical loading and maintain better stability while compromising the minimally invasive plan. Therefore, DPF can be improved to better reduce the stress concentration of the steel plate.

DPF using a volar plate induced lower stress on the calcaneus than TPF, but higher stress on the implant. This could be due to the principle of load transfer, in which the implants shared and sustained load from the fractured bone. The maximum calcaneal stress in the DPF was less than that in the TPF. In addition, the maximum calcaneal stress in the TPF also exceeded the ultimate stress of calcaneal fracture (Frost, 1997), 199.4 *vs.* 130 MPa, which may reduce the likelihood of postoperative rehabilitation exercise. Our study found that stress was concentrated on the middle but slightly anterior portion of the implant, which was consistent with another study (Ouyang et al., 2017). In addition, concentrated stress was observed adjacent to the calcaneal tuberosity in the DPF. The fracture movement will induce high shear on the plate upon weight bearing, which leads to stress concentration. It should be noted that the stress on the volar plate implant during push-off was alarmingly high and exceeded the ultimate strength of titanium (Sitthiseripratip et al., 2003). This indicated that early weight-bearing rehabilitation should be avoided.

Interfragmentary screw fixation is a simple and effective method to enhance fixation stability and could be supplemented with DPF. Wang et al. (1998) compared the biomechanical stability of the lateral plate with and without interfragmentary screws in the treatment of calcaneal fracture and showed that the lateral plate with interfragmentary screws could sustain a higher load before failure. In addition, locking plates were shown to produce higher stiffness but higher peak stress compared to crossing metallic screws (Ni et al., 2016). Ni et al. (2019) compared the stability among fixations using a locking plate, calcanail, and calcanail with interfragmentary screws (modified calcanail system) for DIACFs. Their findings suggested that adding interfragmentary screws to the calcanail system can effectively enhance the fixation stability and reduce the stress concentration. As such, we anticipated that adding interfragmentary screws would also complement the stability of the volar plate system and reduce the risk of implant fatigue, which requires further investigation.

This study had several limitations. Firstly, the fracture line was reproduced according to previous studies, while the form of the actual fracture could be a variant. Future studies may consider implementing a Monte Carlo approach to generate different forms of fracture models according to their incidence of location (Ni et al., 2021). Secondly, the geometry and material properties were simplified in our simulation. For example, the ligaments and fascia were modeled as one-dimensional truss units, while the mechanical loading and behavior of the muscles were also simplified. Reconstructing the three-dimensional geometry of the plantar fascia and ligaments may facilitate a more comprehensive and accurate simulation outcome (Chen et al., 2020; Peng et al., 2021b). The trabecular core and cortical layer of the bone were not segmented to compromise the complexity, size, and number of bones involved in the whole foot-and-ankle complex structure, which was also a common simplification approach in relevant models (Morales-Orcajo et al., 2016). It should be noted that the simplification may have an apparent influence on the strain of the metatarsals (Trabelsi et al., 2014). In addition, we assumed that the bone and soft tissues were homogeneous and isotropic. Future studies may consider implementing anisotropic properties for soft tissues (Aldieri et al., 2018) and wrapping a skin layer (Wong et al., 2020). For the Achilles tendon force, a previous model often disregarded the geometry of the muscle bulk and applied the force directly on the calcaneus (Chen et al., 2012). We attempted to improve the model by accounting for the material properties of those muscles as passive bulk soft tissue. Some literature constructed a specialized fiber matrix component to resemble both the active and passive mechanical behaviors in the FE model (Yucesoy et al., 2002).

A sensitivity analysis may be conducted to address the variance of model assumption (Wong et al., 2018), while the patient-specific muscle force profile could be obtained by a musculoskeletal model to drive the FE model (Peng et al., 2021a). A sensitivity test on the coefficient of friction between bone fragments could enable the quantification of the biomechanical role of the plate. Our model was previously validated by plantar pressure in an intact state only (Zhang et al., 2020). While direct validation of the fracture model with surgical interventions was the ideal case, validation experiments often came with a paradox of the necessity of simulation if physical experiments could be arranged, along with queries on ethics, feasibility, time, and cost. Wong et al. (2021) suggested providing different modalities of evidence and measurement metrics to enhance the model credibility in the case of indirect or scaled model validation. For example, Ni et al. (2016) constructed a partial model of the calcaneus and validated surgery simulation using a cadaveric experiment with a preset loading condition. Model verification is often targeted to quantify the mesh discretization error. In this study, we determined the mesh size based on an existing model that passed the mesh convergence test (Wong et al., 2018) while ensuring the mesh quality by the mesh creation software (Hypermesh). Lastly, our study was confined to a patient-specific single-subject design such that we viewed our model as typical and representative based on the expertise of the orthopedic surgeon (Wong et al., 2021). Future studies could investigate volar plates with supplementary interfragmentary screw fixation to improve fixation stability and alleviate concentrated implant stress.

In conclusion, we proposed a novel surgical option for DPF with a volar plate in treating DIACFs in this study. The biomechanical performance of DPF was compared to that of TPF using the calcaneal plate system through a computational simulation approach (FE method). The results showed that DPF produced less stress on the calcaneus, less displacement of the fragment, and changes in the Böhler angle and Gissane’s angle, which indicated better fixation stability and less risk of stress fractures. Future studies may consider further enhancing the fixation stability and alleviating the concentrated stress at the volar plate by interfragmentary screw fixation.

## Data Availability

The original contributions presented in the study are included in the article/Supplementary Material, further inquiries can be directed to the corresponding author.
